# Supplementary material for: Genetic Correlations Greatly Increase Mutational Robustness and Can Both Reduce and Enhance Evolvability
Source: PLoS Comput Biol. 2016 Mar 3;12(3):e1004773. doi: 10.1371/journal.pcbi.1004773 (PMC4777517; doi:10.1371/journal.pcbi.1004773)
Supplement: S1 Text — (PDF) [file pcbi.1004773.s001.pdf]

Supplementary Material

Associated analytical results of the sampling threshold

We begin with an analysis of the sampling threshold γ from average phenotypes in a given GP map. We showed in Eq. (7) that for effective sampling, we require

$$f_q > \frac{1}{F_p(K-1)L}$$

which may be expressed alternatively as

$$f_p f_q > \frac{1}{K^L(K-1)L}$$

The average phenotype frequency may be written down as

$$\langle f \rangle_{\text{phenotype}} = \frac{1}{N_P}\sum_i f_i = \frac{1}{N_P}$$

or with respect to the genotype sampling distribution as

$$\langle f \rangle_{\text{genotype}} = \sum_i f_i^2$$

Substituting in the smaller of the two, the average phenotype frequency, and then considering the required threshold for effectively sampling phenotype q from the average phenotype p, we find

$$f_q > \frac{N_P}{K^L(K-1)L}$$

For RNA, where empirical scaling values are known ($N_P \approx 1.5 \times L^{-\frac{3}{2}} 1.8^L$ [?]), we can further write

$$f_q \gtrsim 0.45^L \frac{1}{L^{\frac{5}{2}}}$$

for a phenotype q to be effectively sampled.

We can change the question of effective sampling to ask the conditions on N_P for the average phenotype q to be accessed from the average phenotype p. In these circumstances, we can see that

$$N_P < \sqrt{K^L(K-1)L}$$

for which we can see RNA satisfies to an increasing extent for increasing L, as $2^L 3L - 1.93^L$ monotonically increases with increasing L.

Finally, we can also write down the approximate fraction less than the average phenotype frequency that is accessible from an average phenotype, through expressing $f_q = \chi \langle f \rangle_{\text{phenotype}}$ which leads to a threshold fraction

$$\chi = \frac{N_P^2}{K^L(K-1)L}$$

Again, using RNA as an example system this leads to

$$\chi \propto 0.81^L \frac{1}{L^4}$$

for a given length of RNA, showing that an increasing fraction of phenotypes with frequencies below the average may be effectively sampled from the average phenotype.

Extended analysis of ϕ_{qp} across a broader range of phenotypes

In this section, in contrast to the analysis in the main body considering the frequency of phenotypes q around phenotype p where $f_p \ll \gamma$, we consider different p across a range of phenotype frequencies f_p in the GP map. In the random null model, at values of $f_q > \gamma$, we expect phenotypes to almost exactly follow $\phi_{qp} = f_q$. When $f_q < \gamma$, there are two likely possibilities for a given phenotype: either the phenotype is not found at all ($\phi_{qp} = 0$) or it is found a single time ($\phi_{qp} = \gamma$). The latter is an over-representation of the local prevalence of q for the GP map, while the former is clearly no local representation at all.

In Supporting Fig. S1, we display three pairs of plots for the RNA12, $S_{2,8}$ and HP24 GP maps and a randomised null model counterpart. The null models are displayed on the left, with the actual GP maps on the right. In each plot, we show the values of ϕ_{qp} against f_q for three different phenotypes p (coloured by data point as red, blue and green, with red the largest frequency phenotype and blue the smallest). Upward triangular data points represent values for ϕ_{pp}, downward triangular data points $\phi_{qp} = 0$ (shown at 1e-8 for visualisation purposes only) and the circular data points are all other phenotypes. Vertical and horizontal dotted coloured lines represent $f_q = \gamma$ and $\phi_{qp} = \gamma$ respectively. The diagonal dashed black line is $\phi_{qp} = f_q$, the null expectation for phenotypes with $f_q > \gamma$.

We begin by discussing the behaviour of the null model. The $S_{2,8}$ and HP24 null models provide the extreme cases. For $S_{2,8}$, all phenotypes are highly frequent and have $f_q \gg \gamma$. Consequently, we see that each of the three phenotypes follows the expected trend of $\phi_{qp} = f_q$ to a very high degree of accuracy (Spearman rank correlation coefficient and p-value in the top left). For the HP24 null model, all frequencies are such that $f_q \ll \gamma$. As such, phenotypes that are found locally are found only once ($\phi_{qp} = \gamma$) and most are not found at all (the many downward triangular points). For the RNA12 null model, the frequency of phenotypes used for phenotype p span the range of all $f_q \gg \gamma$ (red and green) and to some phenotypes having $f_q \approx \gamma$ (blue). As a result, we see the red and green phenotypes follow $\phi_{qp} = f_q$ strongly, while the tail of the blue phenotype has fluctuations between the three behaviours.

The results from the null models demonstrate the accuracy of the above outlined intuition for a null relationship between the local connectivities of phenotypes with respect to the global abundance. With this in mind, we can now draw direct comparisons between each phenotype in the null model and actual behaviour exhibited in the biological GP maps. For each of the GP maps, we plot the same phenotype as in the null model case. For RNA12, positive correlations are found for each phenotype, with deviations from $\phi_{qp} = f_q$ being more pronounced for lower frequency phenotypes (blue is subject to much greater fluctuations than red). The fluctuations

are approximately up to an order of magnitude either side of the $\phi_{qp} = f_q$. We see a similar behaviour for $S_{2,8}$, with the largest fluctuations exhibited for the low frequency blue phenotype.

Finally, we consider the biological HP24 GP map. As was the case in the null model version, every phenotype (apart from the deleterious phenotype) lies in the region where $f_q \ll \gamma$. The notable difference in the actual GP map is the tendency for phenotypes with a larger f_q to also be more likely to be locally present ($\log \phi_{qp} \propto \log f_q$). We can understand this with the following rationale: due to the neutral correlations present, if a single genotype with phenotype q is found locally, then it is also likely that other genotypes with phenotype q will be local to genotypes with phenotype p. And due to this effect being more pronounced for phenotypes with a greater frequency ($\rho_p \propto \log f_p$, c.f. Fig. 2), we also see this effect locally with increased f_q resulting in a greater ϕ_{qp}, leading to the positive proportionality between $\log \phi_{qp}$ and $\log f_q$ in the actual GP maps when compared to the null models.

References

[1] Schuster P, Fontana W, Stadler PF, Hofacker IL. From Sequences to Shapes and Back: A Case Study in RNA Secondary Structures. Proceedings of the Royal Society of London B: Biological Sciences. 1994;255(1344):279–284.
