# Supplementary figures and images for: Genetic Correlations Greatly Increase Mutational Robustness and Can Both Reduce and Enhance Evolvability
Source: PLoS Comput Biol. 2016 Mar 3;12(3):e1004773. doi: 10.1371/journal.pcbi.1004773 (PMC4777517; doi:10.1371/journal.pcbi.1004773)

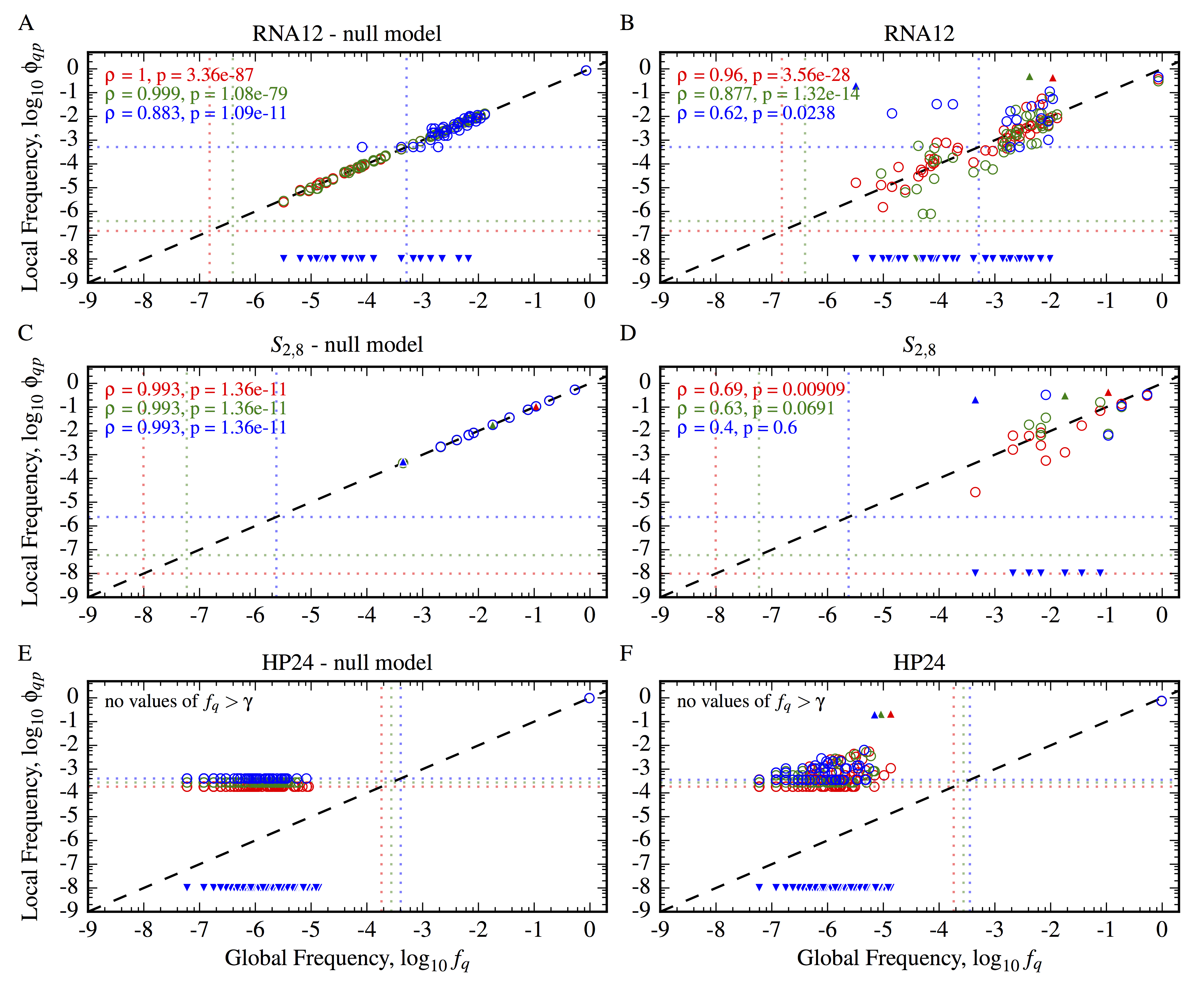

Supplement: S1 Fig — Local frequency of phenotypes q around genotypes with phenotype p (ϕqp) are plotted against the frequency of phenotypes q (fq) for each biological GP map and a random null model counterpart. The black dashed line is ϕqp = fq. The dotted lines are ϕqp = γ and fq = λ (c.f. Eq (7)). In each case, three different phenotypes p with different frequencies in the GP map are considered (represented with red, green and blue from largest to smallest fq). (A, C and E) We plot local against global frequency for the random null models. S2,8 and HP24 illustrate the two regimes where fq > γ and fq < γ in all cases respectively. The former has local frequencies strongly determined by global frequency (ϕqp = fq), while in the latter, occurrences of phenotypes are rare; they may not occur at all (downward triangular points, ϕqp = 0) or they simply occur a single time (ϕqp = λ). In the RNA12 null model, we see the blue phenotype crossing the threshold with some phenotypes having fq ≈ λ. (B, D, F) The three phenotypes are considered in each biological GP map. For larger frequency phenotypes (red and green in RNA12 and S2,8), we find that local frequency is, to first order, well determined by the global frequency in line with the random null models (up to an order of magnitude variation in local frequency in comparison to global frequency). For lower frequency phenotypes (blue in RNA12 and S2,8), we see that phenotype correlations are more important, an intuitive result given the genotypes of p will be less encompassing of the whole GP map in these cases. In HP24 all frequencies are well below the gamma threshold but we still see a positive (although weaker) relationship between local frequency and global frequency (unlike in the null model, where ϕqp remains flat with respect to fq for fq < λ). This is due to the presence of neutral correlations, an effect discussed in greater detail in the main text. (TIFF) [file pcbi.1004773.s002.tiff]
